# Case report: Efficacy and safety of regorafenib plus fluorouracil combination therapy in the treatment of refractory metastatic colorectal cancer

**DOI:** 10.3389/fonc.2022.992455

**Published:** 2022-12-15

**Authors:** Emaan Haque, Ibrahim N. Muhsen, Abdullah Esmail, Godsfavour Umoru, Charisma Mylavarapu, Veronica B. Ajewole, Maen Abdelrahim

**Affiliations:** ^1^ College of Medicine, Alfaisal University, Riyadh, Saudi Arabia; ^2^ Department of Medicine, Houston Methodist Hospital, Houston, TX, United States; ^3^ Section of Gastrointestinal Oncology, Houston Methodist Neal Cancer Center, Houston, TX, United States; ^4^ Department of Pharmacy, Houston Methodist Hospital, Houston, TX, United States; ^5^ College of Pharmacy and Health Sciences, Texas Southern University, Houston, TX, United States; ^6^ Cockrell Center for Advanced Therapeutic Phase I program, Houston Methodist Research Institute, Houston, TX, United States; ^7^ Department of Medicine, Weill Cornell Medical College, New York, NY, United States

**Keywords:** regorafenib, 5-FU, metastatic colorectal cancer, CRC, fluorouracil (3385)

## Abstract

**Background:**

More than half of patients with colorectal cancer (CRC) present with metastatic disease or develop recurrent disease on first-line and second-line options. Treatment beyond the second line remains an area of unmet need for patients with progressive or recurrent disease.

**Methods:**

We retrospectively reviewed data of adult (>18 years old) patients with mCRC who received regorafenib + 5FU combination therapy at Houston Methodist Hospital with outcomes of interest including response rate, discontinuation due to side effects, and overall survival.

**Results:**

Seven patients received regorafenib + 5FU combination therapy for mCRC after receiving at least two other lines of therapy (including at least one fluorouracil-based therapy). Four patients (57%) achieved disease control in 7-12 weeks after therapy initiation while three patients developed recurrent disease. In patients who achieved disease control, no new adverse events were reported among patients with this combination.

**Conclusion:**

Regorafenib and Fluorouracil combination could be considered an option beyond the second line for patients with treatment-refractory metastatic colorectal cancer. Further studies, including a prospective trial, are needed to investigate the efficacy and safety of regorafenib plus 5FU therapy compared to other limited available therapies.

## Introduction

In the United States, about 20-40% of patients diagnosed with colorectal cancer go on to eventually develop metastasis (1), and about 21% of patients have metastasis at the time of diagnosis (2). The treatment strategy for metastatic colorectal cancer (mCRC) is to limit primary tumor expansion and prevent further metastasis with chemotherapy and targeted therapy as the mainstay of management (3–5). Multiple regimens are currently being used in mCRC, with first and second-line options consisting of fluoropyrimidine-based therapy in combination with other cytotoxic chemotherapeutic agents. Examples of such regimens include the FOLFOX (5-FU, Folinic acid, oxaliplatin) and FOLFIRI (5-FU, Folinic acid, irinotecan) regimens, which are used in the first-line and second-line settings, respectively (4, 6, 7). However, treatment options beyond the second line of refractory mCRC remain challenging and despite the availability of multiple options, outcomes are generally poor (8–10).

Regorafenib (Stivarga, BAY 73-4506) is a tyrosine kinase inhibitor that can inhibit multiple kinases essential for neoplasm growth (11, 12). This medication was approved by the Food and Drug Administration (FDA) in 2012 (13) as subsequent therapy for the management of mCRC in patients who have progressed on fluoropyrimidine, oxaliplatin, irinotecan-based chemotherapies, anti-VEGF agents such as bevacizumab, and an anti-EGFR (if KRAS wild-type) therapy (14). This approval came after regorafenib showed a modest increase in overall survival (OS) in comparison to placebo in multiple trials (15, 16). Several studies have attempted to combine regorafenib with other agents and have hypothesized that a possible synergistic effect of combining Regorafenib with fluoropyrimidine-based chemotherapy regimens may result in better outcomes. Ma et al. (17) conducted a retrospective study to evaluate the efficacy and safety of regorafenib and concomitant FOLFIRI treatment irinotecan-base irinotecan-based on UGT1A1 genotype for 1 heavily treated follow-up with mCRC. After a median follow-up of 10 months, the study reported a median OS of 12.0 months and a median progression-free survival (PFS) of 6.0 months. Recently, Adenis et al (18) reported a study with full-dose FOLFIRINOX (5-FU, Folinic acid, irinotecan, oxaloplatin) plus full-dose regorafenib (160mg/day, days 4–10) can be safely delivered, those 13 patients have reported an overall response rate of 62% (95% CI 32%-86%) and median PFS was 9.1 months (range: 3.1; 15.4).

In this study, we report our center’s experience with using a combination therapy of regorafenib and intravenous 5-FU (in addition to leucovorin) for the treatment of patients with refractory mCRC.

## Methodology

### Study design

This is a case series of mCRC patients (biopsy-proven adenocarcinoma of the colon or rectum with radiological evidence of metastasis) who were treated with off–label regorafenib in combination with 5-FU in the third-line setting and beyond. We included adult patients (age > 18 years old) who were treated at Houston Methodist Cancer Center between November 2017 and July 2021.

The medical records of all eligible patients were reviewed and evaluated retrospectively for various disease-specific and treatment-specific variables, including demographic information (age, gender, race, etc.), performance status, diagnosis (left-sided vs. right-sided vs. rectal), metastatic sites, mutational data (RAS, BRAF, MSI, etc.), and prior therapies (including surgical intervention and metastasectomy). This study was approved by the institutional review board of the Houston Methodist Research Institute (IRB ID: PRO00031738).

### Clinical outcomes and toxicities

Clinical outcomes of interest included the best response to combination therapy of 5-FU and regorafenib, progression-free survival (PFS), overall survival (OS), and adverse effects. PFS was defined as the time from combination therapy initiation to clinically proven disease progression whereas OS was defined as the time from treatment initiation to death from any cause. We also captured doses of 5-FU and regorafenib at the time of therapy initiation and time of last follow-up or discontinuation. Additionally, notes were reviewed for any documented adverse events, and they were categorized according to the Common Terminology Criteria for Adverse Events (CTCAE) version 5.0.

## Results

### Patients’ characteristics

Seven patients received regorafenib + 5-FU combination therapy for metastatic CRC. Five patients were males and two were females. Ethnically, five of the patients were of Caucasian descent and two were of Asian descent. Patient age ranged from 33-59 years old on diagnosis and from 45-65 years old on therapy initiation. Five patients had left-sided colon cancer, one had right-sided colon cancer and one had rectal cancer. The majority (n=5, 71%) were stage IV on diagnosis and all had a history of resection and were treated with at least three prior lines of therapy. All patients had a mutated KRAS or NRAS except patient # 7 and none had microsatellite instability.

The most common metastasis site was the liver (in all 7 patients) followed by the lung (in 5 patients). Metastasis was confirmed by imaging with CT/PET scans. All patients received fluorouracil, oxaliplatin, and irinotecan-based chemotherapy and an anti-VEGF therapy prior to combination therapy. Additionally, two patients received regorafenib as a monotherapy prior to the use of combination therapy. Other commonly used therapies such as monotherapy or combination therapy included trifluridine/tipiracil, ramucirumab, and capecitabine. Details of therapies used prior to regorafenib + 5-FU combination therapy are listed in **Table 1**.

**Table 1:** Basic characteristics of the included patients.

Patient	Age at therapy initiation (Gender)	Diagnosis (stage at diagnosis)	Chemotherapies prior to Rego+ 5-FU (Including maintenance therapy)	History of tumor resection	Mutations	Sites of metastasis	Comorbidities
**1**	65 (Female)	Left-sided colon cancer (IV)	FOLFOX + Avastin FOLFIRI + Avastin Avastin monotherapy Lonsurf initially with Avastin and then Ramucirumab FOLFIRINOX + Avastin	Yes	KRAS- mutant (G12D) NRAS- neg BRAF- neg MSI-stable	Peritoneal, ovaries, abdominal wall, liver	Osteoarthritis
**2**	48 (Male)	Left-sided colon cancer (III)	Capecitabine+ Oxaliplatin Capecitabine monotherapy FOLFIRI + Avastin mFOLFOX + Oxaliplatin	Yes	KRAS-mutated MYC-mutated TP53-mutated NTRK 1-3- neg	Liver	Hypertension
**3**	58 (Male)	Right-sided colon cancer (IV)	FOLFOX FOLFIRI+ Avastin Restarted FOLFIRI+ Avastin	Yes	KRAS-mutated TP53-mutated PIK3C-mutated BRAF- neg NTRK 1-3- neg MSI-stable HER-2- neg NRAS-neg	Liver and lung	Hypertension Diabetes Mellitus
**4**	46 (Male)	Left-sided colon cancer (II)	FOLFOX CAPEOX + Avastin Capecitabine + Avastin Avastin FOLFOX + Avastin Avastin+ 5-FU FOLFOX + Avastin FOLFIRI + Avastin Lonsurf	Yes	NRAS mutation MSI-stable, FAP with pathogenic APC variant. HER2 negative.	Liver, lungs, bone, and peritoneum	None
**5**	63 (Female)	Left-sided colon cancer (IV)	FOLFOX + Avastin FOLFIRI + Ramucirumab FOLFIRI + ziv aflibercept Lonsurf 5-FU	Yes	NRAS-mutated KRAS-neg BRAF-neg MSI-Stable	Liver and lung	Hypothyroidism Nephropathy
**6**	58 (Male)	Left-sided colon Cancer (IV)	FOLFOX FOLFIRI + Avastin Lonsurf CAPEOX + Avastin	Yes	-BRAF-neg -KRAS-mutated -NRAS-neg -HRAS-neg -MSI-Stable	Liver and lung	Hypertension Hypercholesterolemia
**7**	59 (Male)	Rectal Cancer (IV)	Capecitabine FOLFOX Xeloda + Avastin Irinotecan + Avastin Avastin Panitumumab + Irinotecan Cetuximab + Irinotecan 5FU + Irinotecan + Cetuximab Regorafenib monotherapy	Yes	KRAS wild type MSI stable	Liver and lung	Hypertension Gout

### Clinical outcomes

Disease status was assessed by imaging with CT/PET scans at the start of treatment and routinely during follow-up appointments. Disease control was achieved in four (57.1%) patients. One patient had a partial response (patient # 2) and the other three patients had stable disease on therapy afterward. The best response was achieved 7-12 weeks after the initiation of therapy and two patients (# 1 and # 2) continued to experience disease control for more than 8 months after therapy initiation. Conversely, three patients progressed on therapy 6-8 weeks after initiation and were transitioned to a different therapy. At the time of the last follow-up, which ranged from 2 to 20 months, five out of the seven patients were alive. From a safety perspective, only two out of seven patients (patients # 5 & 7) were transitioned to a different therapy due to reported tolerance. Clinical outcomes of all seven patients are summarized in **Table 2**.

**Table 2:** Outcomes in patients receiving regorafenib and 5-FU.

Patient #	Best response (time to the best response from initiation in weeks)	Progression or discontinuation of therapy (time to progression/therapy discontinuation)	Therapy after Rego+5-FU	Time to the last follow-up (in months)	Status at last follow-up
**1**	Stable disease(7)	No	None	10	Alive on therapy
**2**	Partial response(12)	No	None	8.5	Alive on therapy
**3**	Progressive disease(7)	Progression (7)	Nivolumab + regorafenib TAS102 + Avastin Tolfenamic acid	20	Alive on other therapy
**4**	Progressive disease(8)	Progression (30)	Pembrolizumab+ regorafenib	9.6	Deceased
**5**	Stable disease(10)	Discontinuation	Lonsurf + Avastin	9	Alive on other therapy
**6**	Progressive disease(6)	Progression	FOLFIRI + avastin	6	Alive on other therapy
**7**	Stable disease(7)	Discontinuation	None	2	Deceased

### Dosages used and toxicities

All patients were started on a regorafenib dose of 40-80 mg which was escalated in one to two weeks to a goal dose of 120 mg and up to 160 mg as tolerated (see **Table 1**). Three patients required further dose reductions to 80 mg for tolerability. Only one patient tolerated treatment with 160 mg but subsequently developed pneumonitis. Regarding 5-FU dosage, six patients received 5-FU 400 mg/m^2^ bolus and 5-FU continuous infusion of 2400 mg/m^2^ over 46 hours starting on day 1 of therapy; however, three patients required a 20% dose reduction in 5-FU for tolerability.

Patient # 5 was initially started on 80 mg of regorafenib which was subsequently increased to 120 mg, however, the patient developed grade 3 hand-foot syndrome (HFS) which was switched to another treatment plan. Patient # 7 was started on regorafenib initially as monotherapy before the addition of 5-FU. This patient developed regorafenib-related pneumonitis necessitating home oxygen and discontinuation of combination therapy. The patient improved on a steroid course with taper.

Other patients reported mild adverse effects that led to dose adjustments. For instance, patient #1 developed grade 2 HFS which necessitated regorafenib dose adjustments from the goal dose of 120 mg to 80 mg daily. Further adjustments included holding regorafenib on the days of 5-FU infusion and a 20% dose reduction in 5-FU dosage. Patient #3 had a history of hypertension and had recorded hypertensive urgency with a systolic blood pressure >180 mmHg. The patient’s blood pressure medications were adjusted, and he had no recurrence of hypertensive urgency. The other patient tolerated the medication at the goal dose of 120 mg. Treatment dose and adverse effects for all seven patients are summarized in **Table 3**.

**Table 3:** Dosing and reported side effects for patients receiving regorafenib and 5-FU, *All patients were started at a dose of 40-80 mg for a week and then increased to the dose listed.

Patient #	Regorafenib dose at initiation *	Regorafenib dose at last follow-up or discontinuation	5-FU dose at initiation (mg/m^2^)	5-FU dose at last follow-up or discontinuation (mg/m^2^)	Side effects reported
**1**	120 mg	80 mg	Day 1: 400 Day 2: 2400	Day 1: 320 Day 2: 1920	Grade 1-2 hand-foot syndrome
**2**	80 mg	120 mg	Received in an outside facility	Received in an outside facility	Well-tolerated
**3**	120 mg	120 mg	Day 1: 400 Day 2: 2400	Day 1: 400 Day 2: 2400	Grade 3 hypertension
**4**	120 mg	120 mg	Day 1: 400 Day 2: 2400	Day 1: 400 Day 2: 2400	Well-tolerated
**5**	120 mg	80 mg	Day 1: 400 Day 2: 2400	Day 1: 320 Day 2: 1920	Grade 3 hand-foot syndrome
**6**	120 mg	80 mg	Day 1: 400 Day 2: 2400	Day 1: 320 Day 2: 1920	Grade 1-2 mucositis
**7**	160 mg	160 mg	Day 1: 320 Day 2: 1920	Day 1: 320 Day 2: 1920	Grade 3 pneumonitis

## Discussion

The metastatic spread of colorectal cancer is generally associated with a poor prognosis, and chemotherapy is currently the mainstay of management for this disease (3, 4). At this time, although there are many alternate rescue therapies to regorafenib for the treatment of mCRC beyond the second line, there is no standardized approach to utilize all the available medications (19). Establishing the biomarker and molecular profile of the tumor is essential for choosing therapies that can effectively target the patient’s individual tumor mutations. For example, tumors with high microsatellite instability can be targeted with pembrolizumab or patients who test positive for NRTK fusions can be targeted with the NRTK inhibitor larorectinib (19, 20). It should be noted that screening for defective DNA mismatch repair using immunohistochemistry and/or microsatellite instability tests still poses many challenges due to difficulties in distilling the biological and technical heterogeneity of microsatellite instability testing into usable data. It has been reported that immunohistochemistry testing of the mismatch repair machinery may give different results for a given germline mutation which suggests the role of somatic missense mutations in mismatch repair immunohistochemistry (21).

Nevertheless, although this personalized approach to treat refractory mCRC can be used in a certain subset of patients, currently the only FDA-approved standardized treatment strategy for mCRC beyond the second line is the use of regorafenib or the combined agent trifluridine/tipiracil (19). Regorafenib, as discussed in this paper, has considerable beneficial efficacy for patients with refractory mCRC beyond the second and third-line, particularly when combined with fluoropyrimidine based therapies. Clinical trial data from the RECOURSE (22) and TERRA (23) study, which evaluated efficacy of trifluridine/tipiracil in patients with mCRC who had been previously treated with fluoropyrimidine-based therapy, showed similar efficacy outcomes, including OS and PFS, as regorafenib in the CONCUR (24) and CORRECT (15) trial. However, since trifluridine/tipiracil is a cytotoxic agent whereas regorafenib is a cytostatic agent, the adverse effect profile for trifluridine/tipiracil is worse and hence, in clinical practice, is usually given after patients have deteriorated on regorafenib (19).

Regorafenib is a multi-kinase inhibitor that targets tumor angiogenesis, oncogenesis, and factors that lead to maintenance of the tumor microenvironment (11, 15). FDA approval was based on the results of the phase III CORRECT trial in mCRC patients which showed a significant improvement in mOS in patients taking regorafenib compared to placebo (15). The results of the CORRECT trial were further supported by the CONCUR trial, a similar phase III trial that took place in various Eastern Asian countries and yielded similar results (24).

In this brief case series, we present seven clinical cases of heavily pre-treated patients with treatment-refractory mCRC who received combination therapy of regorafenib and 5-FU, including one patient who was maintained on therapy for 10 months. Four patients responded to the combination treatment: three patients achieved stable disease, one had a partial response, and one had a mixed response.

Our experience is concordant with recently published clinical data demonstrating the possible efficacy of 5-FU and regorafenib combination in treatment-refractory mCRC patients, as well as other reports illustrating the benefit of regorafenib and 5-FU based therapy (17). For example, Wang et al. (25) described a treatment-refractory mCRC patient who showed partial response in distant metastasis after being placed on fourth-line management with a combination of FOLFIRI (with irinotecan dose escalation) plus Regorafenib. Additionally, Marks et al. (26) described two treatment-refractory mCRC patients who also had a beneficial response after receiving a combination of regorafenib with either 5-FU or capecitabine after failing previous lines of treatment. Results reported that “patient 1” developed the stable disease before passing away after one month of treatment (due to causes unknown to be drug-related) and “patient 2” had stable disease for two months before progressing to develop new metastatic foci. Marks et al. also conducted *in vitro* studies with multiple human colorectal cancer cell lines and determined that there were synergistic effects seen when Regorafenib was combined with 5-FU. Lin et al. (27) conducted a retrospective study for combination use of chemotherapy (either capecitabine, FOLFOX, FOLFIRI, irinotecan, or oxaliplatin) with regorafenib in patients with refractory mCRC and found a median OS of 10.6 months, 11.3 months, and 10.3 months in the regorafenib + FOLFIRI, regorafenib + capecitabine, and regorafenib monotherapy groups, respectively (no p-value calculated). In contrast to these reports which suggest a possible benefit with combination therapy, the single-arm, phase II CORDIAL trial showed that combination therapy of regorafenib with FOLFOX did not improve outcomes in the first-line setting (28). Sanoff et al. (29) conducted a phase II trial of FOLFIRI with regorafenib compared with FOLFIRI alone in the second-line setting for CRC and found no survival benefit with the addition of regorafenib.

Due to the size and design of our study, we could not conclude any predictors of response to treatment. However, it should be noted that Ma et al. (17) found that left-sided colorectal tumors were associated with superior PFS (2.0 vs 7.0 months, p < 0.0001) and OS (4.0 vs 13.0 months, p < 0.0001). Furthermore, they noted that patients with mutated KRAS were found to have shorter OS than patients with wild-type KRAS (6.0 months vs 14.4 months, p = 0.015) and positive EGFR expression had an inverse correlation with PFS (2.5 vs 14.0 months, p = 0.039). One proposed theory for this phenomenon could be due to the fact that KRAS mutations were significantly associated with positive EGFR expression (p = 0.026) and EGFR-positive patients have been shown to have a poor response to FOLFIRI in other studies (15). Five patients in our study had left-sided cancer and only one patient had a right-sided tumor. Thus, we cannot make any definite conclusions about whether left-sided versus right-sided tumors had a better response to combination therapy, or whether this combination would be effective in EGFR positive and mutated KRAS patients. However, these factors should be explored further in future studies as predictors of tumor response in combination therapy of regorafenib with fluoropyrimidine-based therapy. Additionally, several studies have highlighted the potential utility of miRNAs as biomarkers in either tissues or blood for the assessment of response to EGFR inhibitors such as Regorafenib. For example, overexpression of miR-31, miR-100, and miR125b are correlated with resistance to cetuximab. Development of miRNA signature for predicting treatment response designates a personalized therapeutic approach to colorectal cancer (30). Although we did not use miRNA to assess treatment response in our patient cohort, this strategy can also potentially be used in future studies.

In our patient cohort, the majority of adverse events associated with the combination of 5-FU and regorafenib were low grade, and five out of seven patients required clinically appropriate regorafenib dose modifications. Of note, two patients stopped treatment due to intolerable adverse effects. However, these findings are similar to those seen in the phase II CORDIAL trial which reported that 96.2% of their patients required regorafenib dose modifications (either reductions or interruptions) as a result of regorafenib-related adverse events; most commonly diarrhea and hand-foot skin reactions, with four patients dropping out due to treatment-related adverse effects (28). It should be noted that in the CORDIAL trial, all patients were initially started on the recommended regorafenib dose of 160 mg, whereas in our patient cohort all but one patient received a regorafenib goal dose of 120 mg. Instead of the recommended regorafenib dose of 160 mg, we applied a dose-escalation–based strategy to a maximum dose of 120 mg. Our rationale for this was based on findings from the ReDOS trial (31) which provided evidence for optimization of regorafenib dosing and suggested a lower incidence of toxicity with this approach. In addition, the pharmacodynamics of regorafenib at 120 mg was comparable to 160 mg in the phase 1 trial. In practice, a longer duration of therapy on regorafenib may be more important than escalating to the recommended 160 mg. Given the poor performance status that is often associated with this heavily pre-treated patient population, we opine that our dose-escalation strategy was critical to the favorable toxicity profile we observed. Our toxicity profile was also consistent with a phase 1b trial conducted by Schultheis et al. (32) which demonstrated acceptable tolerability and pharmacokinetic interaction when giving regorafenib sequentially after cytotoxic chemotherapy (FOLFOX or FOLFIRI) in mCRC patients. Limitations of this case series include the retrospective nature and the number of evaluable patients at a single academic medical center.

## Conclusion

Our data illustrate the potential clinical benefits of regorafenib plus 5-FU combination beyond the second or third lines of treatment for mCRC patients. Consequently, the efficacy and safety of regorafenib combination therapy with 5-FU as a potential option beyond the second line should be prospectively evaluated and compared to other salvage therapies.

## Data availability statement

The raw data supporting the conclusions of this article will be made available by the authors, without undue reservation.

## Ethics statement

The studies involving human participants were reviewed and approved by Institutional review board statement: The study was conducted according to the guidelines of the Declaration of Helsinki and approved by the Institutional Review Board of Houston Methodist Hospital. The patients/participants provided their written informed consent to participate in this study.

## Author contributions

EH: writing (original draft), visualization, IM: conceptualization, writing (original draft), writing (review & editing), data curation, visualization, AE: writing (review & editing), final draft (review & editing), data curation, visualization, and critical review, CM: writing (review & editing), data curation, VA: writing (review & editing), data curation, GU: writing (review & editing), data curation, MA: conceptualization, project administration, supervision, validation, visualization. All authors contributed to the article and approved the submitted version.
